# New Medical Schools

**Published:** 1852-10

**Authors:** 


### NEW MEDICAL SCHOOLS.

We owe an apology to our friends of the new Medical School of Cincinnati for not noticing their organization in our last number. It is styled the "*Miami Medical College of Cincinnati,*" and Dr. Mussey, late of the Medical College of Ohio, is at the head of it. It would appear from this that the venerable and distinguished surgeon was not so weary of teaching as some of his friends supposed he had grown. His colleagues are Drs. Judkins, Avery, Davis, White, Mendenhall, Murphy, Comegys, and John Locke, Jr. We heartily wish them success in their enterprise, assured as we are that their institution will be conducted upon principles honorable to themselves and to the profession.

And we have to record the incorporation of still another medical college—the *Savannah Medical Institute.* This is the second medical school for Georgia. The gentlemen composing the faculty are Drs. Arnold, Kollock, Bulloch, Byrd, Howard, Martin, West, and Read; "young, ardent, energetic" men, we learn from the Charleston Medical Journal, of whom the editor remarks that, "with a reputation to earn, there is no reason to fear that they will prove to be laggards in the race for professorial as well as professional distinction."

